# Effect of Wenxin Granule on Ventricular Remodeling and Myocardial Apoptosis in Rats with Myocardial Infarction

**DOI:** 10.1155/2013/967986

**Published:** 2013-08-13

**Authors:** Aiming Wu, Jianying Zhai, Dongmei Zhang, Lixia Lou, Haiyan Zhu, Yonghong Gao, Limin Chai, Yanwei Xing, Xiying Lv, Lingqun Zhu, Mingjing Zhao, Shuoren Wang

**Affiliations:** ^1^Key Laboratory of Chinese Internal Medicine of Ministry of Education and Beijing, Dongzhimen Hospital Affiliated to Beijing University of Chinese Medicine, Beijing 100700, China; ^2^National Engineering Research Center for R&D of TCM Multi-Ingredient Drugs, Beijing 100079, China; ^3^Beijing University of Chinese Medicine Institute for Cardiovascular Disease, Dongzhimen Hospital Affiliated to Beijing University of Chinese Medicine, Beijing 100700, China; ^4^Guang'An Men Hospital, Chinese Academy of Chinese Medical Sciences, Beijing 100053, China

## Abstract

*Aim*. To determine the effect of a Chinese herbal compound named Wenxin Granule on ventricular remodeling and myocardial apoptosis in rats with myocardial infarction (MI). *Methods*. Male Sprague-Dawley (SD) rats were randomly divided into four groups: the control group, the model group, the metoprolol group, and the Wenxin Granule group (WXKL group) with sample size (*n*) of 7 rats in each group. An MI model was established in all rats by occlusion of the left anterior descending coronary artery (the control group was without occlusion). Wenxin Granule (1.35 g/kg/day), metoprolol (12 mg/kg/day), and distilled water (5 mL/kg/day for the control and model groups) were administered orally for 4 weeks. Ultrasonic echocardiography was used to examine cardiac structural and functional parameters. Myocardial histopathological changes were observed using haematoxylin and eosin (H&E) dyeing. Myocardial apoptosis was detected by terminal deoxynucleotidyl transferase mediated dUTP nick end labeling (TUNEL) staining. Serum angiotensin II (Ang II) concentration was measured using the enzyme-linked immunosorbent assay (ELISA). *Results*. It was found that Wenxin Granule could partially reverse ventricular remodeling, improve heart function, alleviate the histopathological damage, inhibit myocardial apoptosis, and reduce Ang II concentration in rats with MI. *Conclusions*. The results of the current study suggest that Wenxin Granule may be a potential alternative and complementary medicine for the treatment of MI.

## 1. Introduction

Myocardial infarction (MI) is an acute and critical disease of the cardiovascular system endangering human health [1]. The prevalence of MI continues to increase in a Japanese population [2]. With the impact of the aging Chinese population, the accelerated pace of modern life, changes in eating habits, and social, psychological, and other factors, the incidence of MI in China shows an increasing tendency [3]. The negative impact on family and society is serious due to the economic and death burdens resulting from MI. Prevention and control of this increased occurrence of MI in the Chinese population is currently unsatisfactory [4]. Currently, the reperfusion therapy such as percutaneous coronary intervention (PCI) has been widely carried out [5], with the early mortality in patients with MI significantly reduced [6]. Whilst a large number of patients with MI survive into the recovery phase, patients are still confronted with the risks of recurrent acute cardiovascular events, readmission to hospital, and unfavorable quality of life [7, 8]. In addition, patients also need to manage difficulties such as severe left ventricular dysfunction and potentially the development of heart failure [9].

Ventricular remodeling and myocardial apoptosis are the primary causes of heart failure following MI and the major pathological factors affecting prognosis of heart failure following MI [10, 11]. Neither ventricular remodeling nor myocardial apoptosis, however, is independent disease. Both diseases are the secondary pathophysiological response process following MI. Ventricular remodeling is the result of overall ventricular compensation represented by a series of changes in heart size, shape, wall thickness, cardiac tissue structure, and aggravation of heart function [12]. Apoptosis, also known as programmed cell death, is a physiological phenomenon. The increase in myocardial apoptosis following MI is one of many mechanisms involved in aggravated cardiac tissue injury [13]. The ventricular remodeling and myocardial apoptosis following MI are inextricably linked with each other [14, 15]. Excessive apoptosis may result in two different events. Whilst excessive apoptosis can accelerate the loss of myocardial cells, deteriorate heart function, and promote the development of ventricular remodeling [10], the ventricular remodeling can also aggravate myocardial ischemia and the excessive apoptosis result in hypoxic injury [16]. Regardless in both cases, ventricular remodeling and myocardial apoptosis are the primary reasons leading to heart failure following MI and ultimately death [17]. Therefore, both reverse ventricular remodeling and inhibition of myocardial apoptosis are beneficial to delay the incidence of heart failure after MI and reduce mortality in patients [18, 19].

Wenxin Granule is a Chinese herbal compound developed by the China Academy of Chinese Medical Sciences and funded by Chinese national “85” science and technology research project. It contains *Radix Codonopsis Pilosulae, Rhizoma Polygonati, Radix Notoginseng, Succinum *and* Radix et Rhizoma Nardostachyos*. In recent years, more and more clinicians have successfully applied Wenxin Granule in cardiovascular disease prevention and treatment, and have received a satisfactory clinical outcome [20–22]. Nevertheless, the role of Wenxin Granule in cardiovascular diseases requires further clinical evidence and definitive mechanisms of action. The current study focused on ventricular remodeling and myocardial apoptosis after MI, in an attempt to provide experimental evidence of the cardioprotective effect of Wenxin Granule in a rat model of MI.

## 2. Materials and Methods

### 2.1. Animals

Male Sprague-Dawley (SD) rats (190–210 g) were purchased from the animal laboratory of the Academy of Medical Sciences, Beijing, China (certificate number SCXK (Beijing) 2009-0007).

### 2.2. Drugs and Reagents

Wenxin Granule was produced by Shandong Buchang pharmaceutical Co., Ltd., Xi'An, China (Med-drug permit number Z10950026, China). According to the Chinese National Pharmacopoeia (National Pharmacopoeia Committee, 2010), the total amount of notoginseng saponin R1 (C47H80O18), ginseng saponin Rg1 (C42H72O15), and ginseng saponin Rb1 (C54H92O23) should not be less than 17.0 mg per bag (5 g). Metoprolol tartrate tablets were produced by AstraZeneca Pharmaceutical Co., Ltd., Jiangsu, China (Med-drug permit number H32025391, China). The terminal deoxynucleotidyl transferase mediated dUTP nick end labeling (TUNEL) apoptosis assay kit was purchased from Wuhan Boster Bio-Engineering limited company (product number MK1020, China). Rat angiotensin II (Ang II) enzyme-linked immunosorbent assay (ELISA) Kit (batch number 201211) was provided by Beijing UBIO Biotechnology Co., Ltd., China.

### 2.3. Establishment of the Myocardial Infarction (MI) Rat Model [23]

Male SD rats were anaesthetised by intraperitoneal (i.p) injection of a 1% solution of sodium pentobarbital (50 mg/kg). The procedures performed consisted of endotracheal intubation; positive pressure ventilation; preoperative recording by twelve-lead electrocardiogram (ECG); one-lead monitoring; local skin disinfection; chest opening; thoracotomy device setup and opening of the pericardium; occlusion of the left anterior descending coronary artery at the location between the pulmonary cone and the left atrial appendage under its origin 2-3 mm. In the control group, the left anterior descending artery was not occluded. Additional twelve-lead ECG recordings were made postoperatively. Successful ligation was confirmed by ST segment elevation in postoperative ECG, compared with preoperative ones. After the coronary artery occlusion surgery, all animals were given penicillin by i.p injection for three days to prevent infection. One rat died due to surgical bleeding during the operation. Within 24 h after surgery, three rats died of ventricular fibrillation following acute MI. The rat mortality rate was 12.5%.

### 2.4. Design and Allocation of Rats

All rats used in this study received humane care in compliance with the National Institutes of Health Guide for the Care and Use of Laboratory Animals. Rats were randomly divided into 4 groups: control, model, metoprolol, and Wenxin Granule (WXKL) groups with each group consisting of 7 rats. The rats in the WXKL group were administrated oral doses of 1.35 g/kg of Wenxin Granule per day. Rats in the metoprolol group were treated with 12 mg/kg of metoprolol tartrate tablets per day. The Wenxin Granule and metoprolol tartrate tablets were grinded and then mixed with distilled water prior to administration. Rats in the control and the model groups were administrated equivalent amounts of distilled water orally each day. The day after the coronary artery occlusion, all rats were administrated treatment orally for 4 weeks. After 4 weeks of treatment, rats were injected with 1% solution of sodium pentobarbital (40 mg/kg), and an echocardiography was performed. Blood samples were taken from the abdominal aorta. After separation of serum, Ang II was determined using an ELISA assay. The heart was excised and weighed for calculation of the heart weight/body weight ratio. The heart samples were fixed in 4% paraformaldehyde for further pathological experiments.

### 2.5. Echocardiography

At 4 weeks after the coronary artery occlusion surgery, a noninvasive transthoracic echocardiography method was used to evaluate the structure and function of the left ventricle in each group. Under anesthesia by i.p injection of 40 mg/kg pentobarbital sodium, rats were fixed on their backs with their fur shaved and skin cleaned. The parasternal long axis view was selected by using a high-frequency linear-array transducer. Then, the parameters of heart structure and function were checked in the two-dimensional ultrasound-guided M-curve. The parameters were automatically recorded and consisted of left ventricular posterior wall end-diastolic thickness (LVPWTd); left ventricular posterior wall end-systolic thickness (LVPWTs); interventricular septum end-diastolic thickness (IVSTd); interventricular septum end-systolic thickness (IVSTs); left ventricular end-diastolic inner diameter (LViDd); left ventricular end-systolic inner diameter (LViDs); end-diastolic volume (EDV); end-systolic volume (ESV); stroke volume (SV); ejection fraction (EF); and fractional shortening (FS). The instrument used was a Sino-Japanese joint AloCa5000 color ultrasound diagnostic apparatus. Echocardiography was operated by a technician who was blind to the grouping allocation.

### 2.6. Myocardial Histopathology

Rat heart samples were fixed in 4% paraformaldehyde and embedded in paraffin. The tissue slices (4 **μ**m) of the heart underwent haematoxylin and eosin (H&E) staining. Histopathological changes were examined and photographed under a light microscope (×400). The experiment of histopathology has been previously described [24].

### 2.7. Apoptosis Detection

Myocardial apoptosis was detected by the method of TUNEL. All procedures were performed as per the manufacturers' instructions. Diaminobenzidine (DAB) was used to label the nucleus. Samples were counterstained with hematoxilin. The nuclei were defined as apoptotic if the whole nuclear area of the cell was labeled positively. The apoptotic cells were counted manually in 5 high-power fields (×400 magnification) by the Image Pro Plus 6.0 program. The apoptosis rate was calculated manually as the percentage of positively staining cells: apoptosis rate = number of apoptotic cells/total number of nucleated cells [25].

### 2.8. Detection of Ang II

Blood samples were taken from the abdominal aorta at 4 weeks after the coronary artery occlusion surgery. The serum was separated by centrifugation at 3,000 rpm for 10 min. The serum was kept at −70°C. The serum was examined using the ELISA method to detect the levels of Ang II. The serum was further analyzed to quantify the concentration of Ang II in strict accordance with the manufacturers' protocols. The main assay procedures are as follows: (1) dilute standard; (2) inject samples and standard wells; (3) add both Ang II-antibody and Streptavidin-HRP, seal the sealing membrane, gently shake, and incubate for 60 min at 37°C; (4) remove the membrane carefully, drain the liquid, and shake away the remaining water; (5) add chromogen solution A and then chromogen solution B to each well. Gently mixed, incubate for 10 min at 37°C in the dark; (6) add Stop Solution into each well to stop the reaction; (7) take blank well as zero, measure the optical density (OD) under the wavelength of 450 nm by using Thermo scientific Multiskan MK3 microplate reader.

### 2.9. Statistical Analysis

Data were analyzed using Statistical Package for Social Sciences (SPSS) for windows (version 13.0). The measurement data were expressed as mean ± standard deviation (SD). The data generated from multiple samples were statistically analyzed by one-way analysis of variance (ANOVA) and Fisher's least significant difference (LSD) test. The count data are expressed as frequency (%). The data from the multiple samples for grade materials were analyzed by the Kruskal-Wallis test. A value of *P* < 0.05 was considered statistically significant.

## 3. Results

### 3.1. The Heart Weight/Body Weight (HW/BW) Ratio and Ventricular Wall Thickness

**Figure 1:**
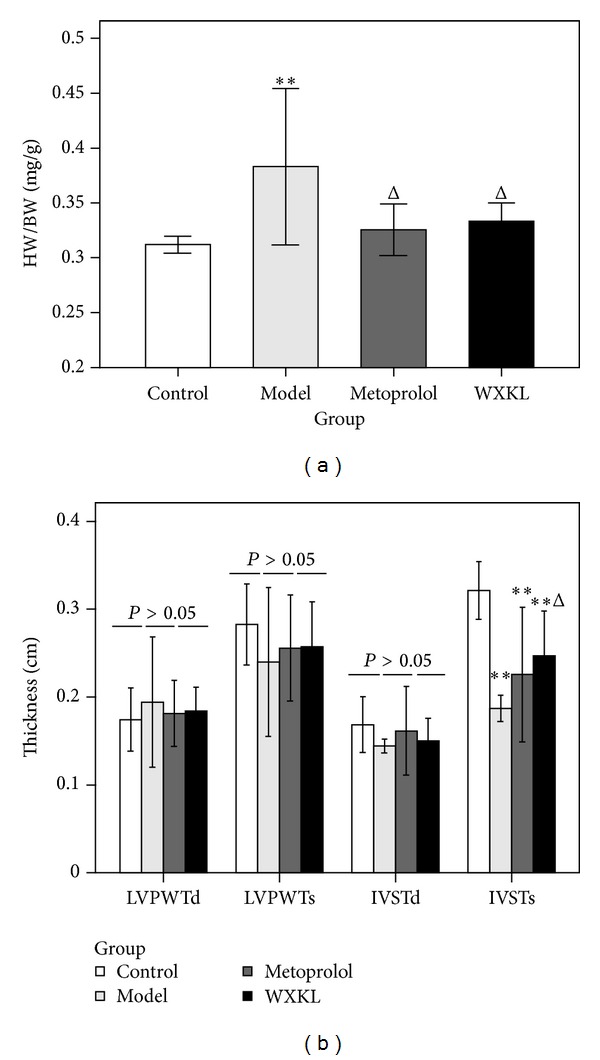
The HW/BW ratio and ventricular wall thickness. The heart weight, body weight, and ventricular wall thickness were detected at 4 weeks after the coronary artery occlusion surgery. (a) The HW/BW ratio. (b) The ventricular wall thickness. Values are expressed as mean ± SD (*n* = 7). **P < 0.01, versus control. ^Δ^ *P* < 0.05, versus model.

As shown in Figure 1(a), the HW/BW ratio in the model group was significantly increased compared with the control group (0.383 ± 0.071 versus 0.312 ± 0.008 mg/g, resp., *P* < 0.01). The HW/BW ratio in the metoprolol group (0.325 ± 0.024 mg/g, *P* < 0.05) and the WXKL group (0.333 ± 0.017 mg/g, *P* < 0.05) was significantly decreased compared with the model group. As shown in Figure 1(b), there was no significant difference between LVPWTd, LVPWTs, and IVSTd among the four groups. The IVSTs in the control group was 0.32 ± 0.03 cm, and those of the model (0.19 ± 0.01 cm, *P* < 0.01), metoprolol (0.23 ± 0.08 cm, *P* < 0.01), and the WXKL groups (0.25 ± 0.05 cm, *P* < 0.01) were significantly lower in various degrees. Compared with the model group, the IVSTs in the WXKL group were significantly increased (*P* < 0.05).

### 3.2. Left Ventricular Contraction Movement, Internal Diameter, and Volume

**Figure 2:**
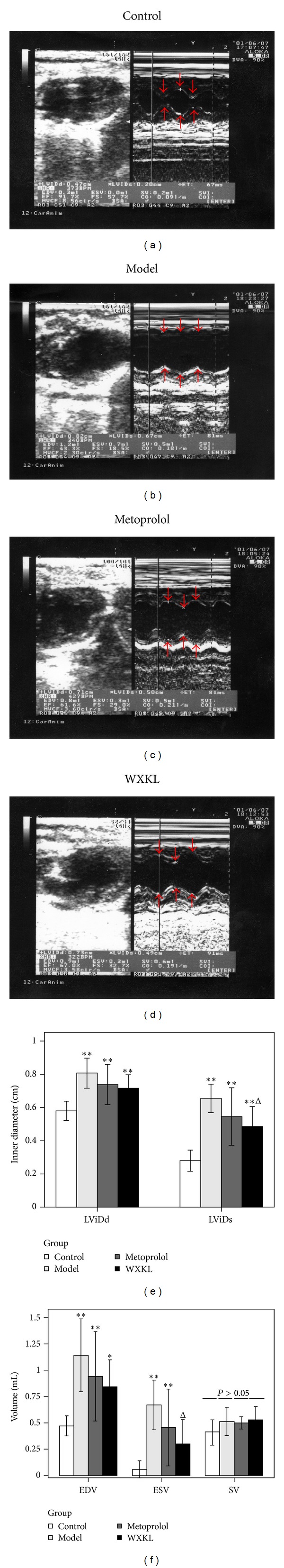
Left ventricular contraction movement, internal diameter, and volume. At 4 weeks after the coronary artery occlusion surgery, echocardiography was performed, and the typical echocardiography images were taken among different groups ((a)–(d)). Internal diameter and volume of the left ventricle show the quantitative analysis data ((e)-(f)). Values are expressed as mean ± SD (*n* = 7). *P < 0.05, **P < 0.01, versus control. ^Δ^ *P* < 0.05, versus model.

At 4 weeks after the coronary artery occlusion surgery, echocardiography was performed, and the typical echocardiography images were taken among different groups. As shown in Figure 2(a), the image of contraction movement in the control group is shaped like waves (red arrow). As shown in Figure 2(b), the waves in the model group weakened and even straightened (red arrow), indicating diminished and even disappearance of contraction movement. The left ventricle expanded significantly in the model group compared with the control group. As shown in Figures 2(c) and 2(d), the changes of left ventricular size and contraction weakness were alleviated in the metoprolol and the WXKL groups. Figures 2(e) and 2(f) show the quantitative analysis of the internal diameter and volume of the left ventricle. The LViDd, LViDs, and EDV in the other three groups were significantly increased to various extents compared with the control group (*P* < 0.01, *P* < 0.05). The ESV in the model and the metoprolol groups also increased significantly (*P* < 0.01). The SV did not show any significant differences between the four groups. Compared with the model group, Wenxin Granule administration decreased LViDs (0.66 ± 0.09 versus 0.49 ± 0.12 cm, *P* < 0.05) and ESV (0.67 ± 0.24 versus 0.30 ± 0.23 mL, *P* < 0.05) but had no effects on LViDd (0.81 ± 0.09 versus 0.72 ± 0.08 cm, *P* > 0.05) and EDV (1.14 ± 0.35 versus 0.84 ± 0.26 mL, *P* > 0.05). Metoprolol had no effects on the left ventricular internal diameter and volume, neither in the systolic nor diastolic phase.

### 3.3. Cardiac Function

**Figure 3:**
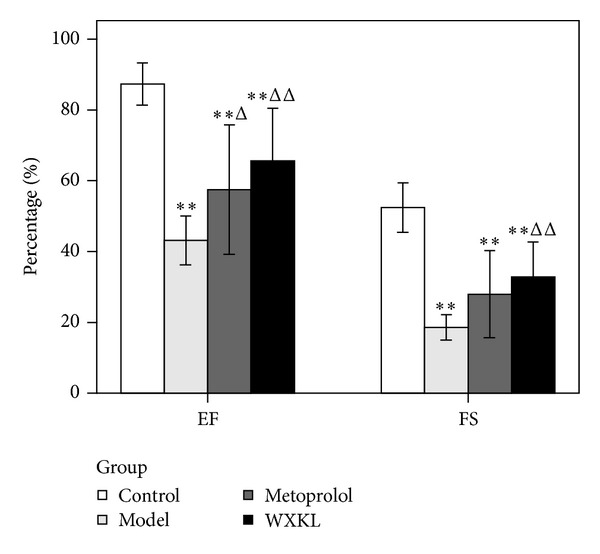
Cardiac function. The EF and FS were detected by echocardiography at 4 weeks after the coronary artery occlusion surgery. The EF and FS were significantly decreased in all groups compared with the control group. Compared with the model group, the EF in the metoprolol group and WXKL group was significantly increased, while only the FS in the WXKL group was significantly increased. Values are expressed as mean ± SD (*n* = 7). **P < 0.01, versus control. ^Δ^ *P* < 0.05, ^ΔΔ^ *P* < 0.01, versus the model.

As shown in Figure 3, the EF and FS were significantly decreased to various extents in the other three groups compared with the control group (*P* < 0.01). The EF in the model group was 43.17 ± 6.89%, and those of the metoprolol group (57.51 ± 18.31%, *P* < 0.05) and the WXKL group (65.67 ± 14.82%, *P* < 0.01) significantly increased compared with the model group. The FS in the WXKL group was significantly increased compared with the model group (32.84 ± 9.85 versus 18.57 ± 3.59%, *P* < 0.01).

### 3.4. Myocardial Histopathological Findings

**Figure 4:**
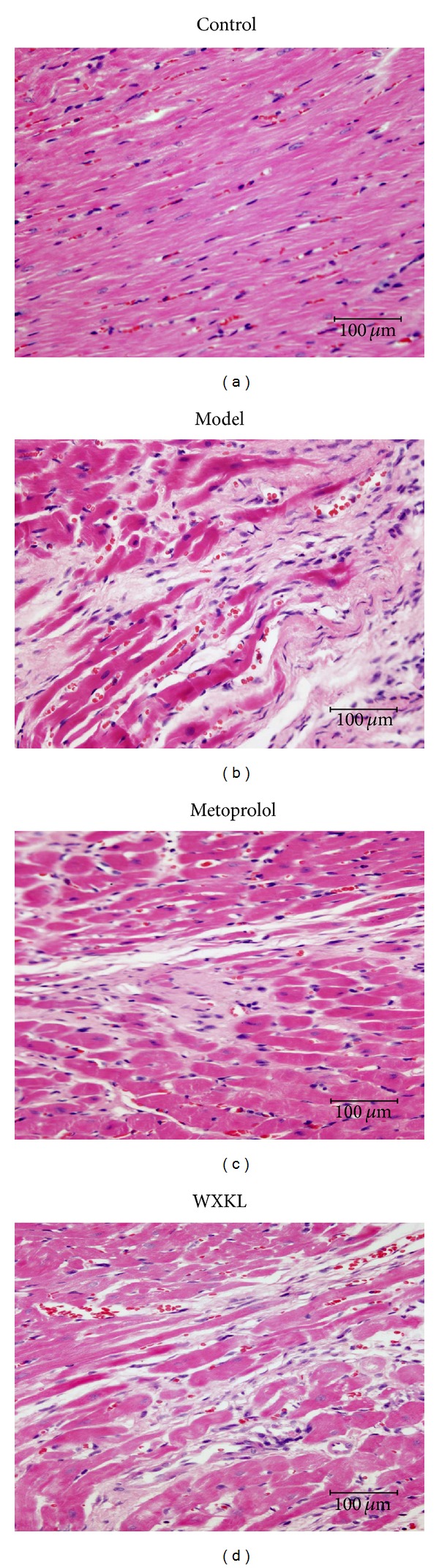
Myocardial histopathological findings. Four-micron-thick sections of myocardial tissue were H&E stained and photographed with a digital camera mounted on a light microscope (×400 magnification; scale bar, 100 *μ*m) at 4 weeks after the coronary artery occlusion surgery. (a) Control group, (b) model group, (c) metoprolol group, and (d) WXKL group.

Myocardial histopathological findings are shown in Figure 4. In the control group, myocardial fibers were arranged in an orderly fashion, cytoplasmic staining was uniform, and nucleus boundaries were clear. In the model group, myocardial fibers arrangement was discorded, numerous neutrophile granulocytes were seen to be infiltrating, and wide range of necrosis observed, while some cytoplasts showed intense staining. Compared with the model group, the previous histopathological changes were alleviated in both the metoprolol and the WXKL groups. According to the literature [24], the severity of necrosis and inflammatory cells infiltration were graded as follows according to staining intensity: −: absent; +: mild; ++: moderate; and +++: severe. As shown in Table 1, the severity of necrosis and inflammatory cells infiltrating in the metoprolol group and the WXKL group were significantly alleviated compared with the model group.

### 3.5. Myocardial Apoptosis

**Figure 5:**
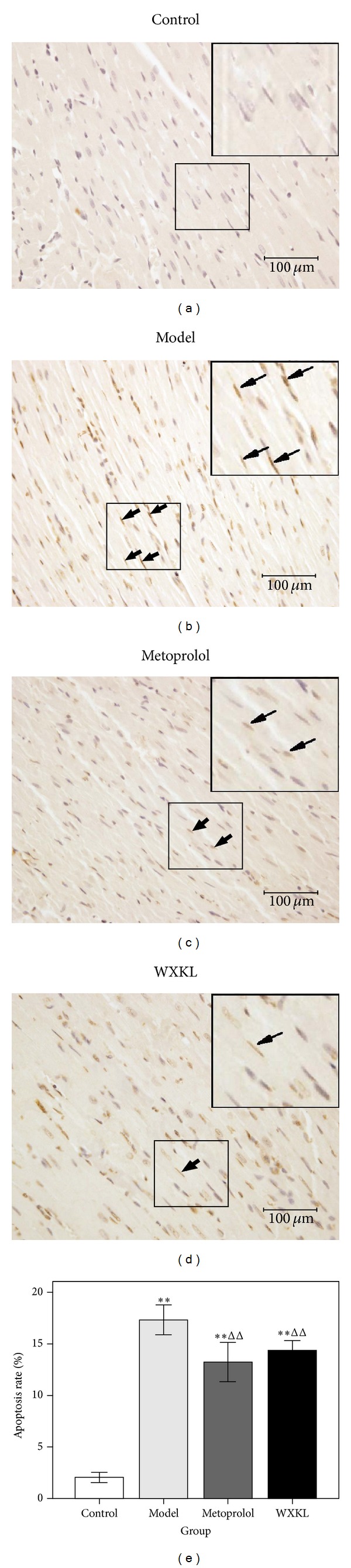
Myocardial apoptosis. Apoptotic nuclei were stained in brownish yellow, while normal nuclei were stained in blue. The typical apoptosis images (TUNEL, DAB; ×400 magnification; scale bar, 100 *μ*m) were taken among different groups (a)–(d). Quantitative analysis of apoptotic rates (e). Values are expressed as mean ± SD (*n* = 7). **P < 0.01, versus control. ^ΔΔ^ *P* < 0.01, versus model.

The technique of TUNEL staining was used to detect myocardial apoptosis at 4 weeks following coronary artery occlusion surgery. Normal nuclei were stained blue, whilst apoptotic nuclei were stained brownish yellow. As shown in Figure 5(a), in the control group, the majority of nuclei were stained blue (normal nuclei), with few nuclei staining brownish yellow (apoptotic nuclei). As shown in Figure 5(b), there was a large number of myocardial apoptotic nuclei in the model group stained brownish yellow. In Figures 5(c) and 5(d), myocardial nuclei apoptosis can be seen to be alleviated in the metoprolol and WXKL groups. The quantitative analysis data of positive staining is shown in Figure 5(e). Compared with the control group, the apoptosis rates were significantly increased at 4 weeks after the coronary artery occlusion surgery. The apoptosis rates in the model group were 17.33 ± 1.46%. Those of the metoprolol group (13.23 ± 1.91%, *P* < 0.01) and the WXKL group (14.36 ± 0.98%, *P* < 0.01) were significantly lower compared with the model group.

### 3.6. Serum Ang II Concentration

**Figure 6:**
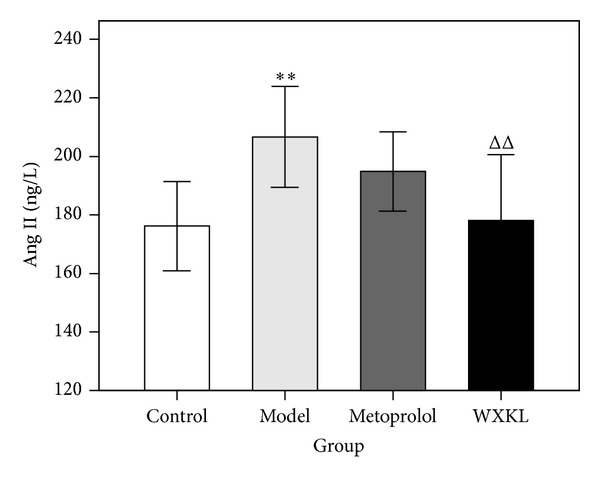
Serum Ang II concentration in the 4 experimental groups. The serum Ang II concentration was detected by ELISA assay at 4 weeks after the coronary artery occlusion surgery. Ang II in the model group was significantly increased compared with the control group. Ang II in the WXKL group was significantly decreased compared with the model group. Values are expressed as mean ± SD (*n* = 7). **P < 0.01, versus control. ^ΔΔ^ *P* < 0.01, versus model.

At 4 weeks after the coronary artery occlusion surgery, the serum Ang II concentration was detected by ELISA assay. As shown in Figure 6, the serum Ang II concentration in the model group was significantly increased compared to the control group (206.62 ± 17.24 versus 176.19 ± 15.24 ng/L, *P* < 0.01). The serum Ang II concentration in the WXKL group was significantly decreased compared with the model group (178.11 ± 22.51 versus 206.62 ± 17.24 ng/L, *P* < 0.01).

## 4. Discussion

In modern society, there has been a growing interest in traditional chinese Medicine (TCM) for patients due to the personalized therapy available in many countries [26, 27]. TCM in cardiovascular disease prevention and treatment is a valuable and promising prospect. TCM has a history of more than 2500 years with a unique theory of diagnosis and treatment. Whilst there is no such term as MI in TCM, its symptoms can be classified into the categories of true heart pain, palpitation, thoracic obstruction, and heart-energy stagnation syndrome. In recent years, an increasing number of clinicians have successfully applied TCM drugs for supplementing Qi, nourishing Yin, and activating blood circulation in the treatment of MI [28, 29]. Nevertheless, the role of TCM in cardiovascular diseases requires further experimental evidence.

Wenxin Granule is a Chinese medicinal granule, which has effects on supplementing Qi and nourishing Yin, promoting blood circulation for removing blood stasis. Several studies have shown that Wenxin Granule can increase coronary blood flow, reduce myocardial oxygen consumption, enhance myocardial compliance, improve myocardial hypoxia tolerance, relieve anterior and posterior cardiac loading, reduce myocardial tissue damage in patients with high blood pressure, and reduce the occurrence of arrhythmia [30]. To further support previous studies, the present study aimed to provide experimental evidence of the cardioprotective effect of Wenxin Granule in the MI rat model.

The results of the current study showed that administration of Wenxin Granule could partially reverse left ventricular remodeling and improve left ventricular function to an extent. It should be noted that whilst the Wenxin Granule decreased LViDs and ESV, Wenxin Granule administration had no effects on LViDd and EDV. It is possible that 4 weeks after modeling, fibrotic scaring or a ventricular aneurysm occurred in the left ventricular infarct wall, which may be responsible for the increase in LViDd and EDV. Wenxin Granule did not completely reverse fibrotic scaring and ventricular aneurysm after MI. Consequently, the LViDd and EDV did not change significantly in the diastolic phase. During the systolic phase, the contractile function of non-infarct myocardium in the Wenxin Granule group was stronger than that in the model group. Therefore, the Wenxin Granule could decrease LViDs and ESV markedly. This was the main difference between the metoprolol group and the WXKL groups whereby metoprolol only showed a decreasing trend on the left ventricular internal diameter and volume, but without significant difference, in either the systolic or the diastolic phase. The possible reasons for this are that the cardioprotective mechanisms of metoprolol are achieved mainly by blocking cardiac *β*1-receptors and thereby slowing heart rate and reduction in myocardial contractility and myocardial oxygen consumption. Compared with other beta-Blockers and angiotensin converting enzyme inhibitors (ACEI), the effect of metoprolol on preventing left ventricular remodeling is relatively weak [31, 32].

In addition, the present study observed the improvement of histopathological injury and the inhibition of myocardial apoptosis after MI in the WXKL group and the metoprolol group. *Radix Notoginseng*, one of the main components of Wenxin Granule, can repair ischemia myocardial and reduce myocardial ischemic injury by decreasing oxidative stress and repressing the inflammatory cascade [33]. This may be the means by which treatment with Wenxin Granule reduced histopathologic injury after MI. Meanwhile, it was also found that Wenxin Granule was capable of reducing Ang II concentrations in the MI rat model. Myocardial ischemia induces activation of various components of the renin-angiotensin system (RAS), including angiotensinogen, renin, angiotensin-converting enzyme, angiotensins, and angiotensin receptors, in the acute phase of MI and in the postinfarction remodeling process [34]. In the RAS, Ang II is a biologically active substance, which is closely correlated with myocardial apoptosis. Several studies have investigated the relationship between Ang II and myocardial apoptosis. A study by Kajstura et al. (1997) showed that in primary cultures of adult rat ventricular myocytes exposed to 10^−9^ M of Ang II for 24 h, presented with a fivefold increase in apoptosis documented by the terminal deoxynucleotidyl transferase assay, and confirmed by DNA agarose gel electrophoresis [35]. A study conducted by Leri et al. (1998) confirmed that Ang II could increase the susceptibility of myocytes to undergo apoptosis [36]. Ang II stimulation was associated with translocation of the epsilon and delta isoforms of protein kinase C. This was coupled with an increase in cytosolic Ca^2+^ in the cells which can induce apoptosis [35]. Several studies have confirmed that* Radix Codonopsis Pilosulae*, one of the main components of Wenxin Granule, can reverse Ca^2+^ influx and the increase in apoptosis. This is achieved by attenuating Ang II and the cardiac-impaired insulin-like growth factor II (IGF II) receptor pathway in myocardial cells [37]. The current study found that treatment with Wenxin Granule decreased Ang II concentrations. Consequently, Ang II could be the underlying mechanism of Wenxin Granule inhibition of apoptosis.

Based on the previous findings, the authors draw the conclusion that Wenxin Granule can partially reverse ventricular remodeling, improve heart function, alleviate the histopathological damage, inhibit myocardial apoptosis, and reduce AngII concentrations in rats with MI. These results suggest that Wenxin Granule might be a potentially alternative and complementary medicine for the treatment of MI.

## Figures and Tables

**Table 1:** The myocardial histopathological changes among different groups (case (%), *n* = 7).

Group	Necrosis				Inflammatory cells infiltrating			
	−	+	++	+++	−	+	++	+++
Control	7 (100%)	0	0	0	7 (100%)	0	0	0
Model	0	1 (14.3%)	4 (57.1%)	2 (28.6%)	0	1 (14.3%)	5 (71.4%)	1 (14.3%)
Metoprolol	0	5 (71.4%)	2 (28.6%)	0	0	1 (14.3%)	6 (85.7%)	0
WXKL	0	4 (57.1%)	3 (42.9%)	0	0	3 (42.9%)	4 (57.1%)	0

*χ* ^2^	19.923				19.475			
*P*	0.000				0.000			
